# General principles of pharmacotherapy selection in elderly patients for different comorbidities

**DOI:** 10.1192/j.eurpsy.2022.152

**Published:** 2022-09-01

**Authors:** M. Stuhec

**Affiliations:** 1 Medical Faculty Maribor Slovenia, Department Of Pharmacology, Maribor, Slovenia; 2 Ormoz Psychiatric Hospital, Clinical Pharmacy, Ormož, Slovenia

**Keywords:** psychopharmacology, Pharmacotherapy, Medication selection, Elderly patients and comorbidities

## Abstract

According to the data, more than 50% of elderly patients with mental disorders have at least one comorbidity and are treated with multiple medications (e.g. 5 or more medications), which can lead to problems in medication selection and medically unnecessary polypharmacy (i.e., irrational polypharmacy). On the other hand, there are still many untreated patients, which can lead to severe disturbances and an excess death rate. Due to frequent comorbidities and treatments in the elderly, medication-related problems are very frequent. Drug-drug interactions (DDIs) between somatic medications and psychotropics often occur in this population. These patients are also excluded from many clinical trials and consequently, this age group is underrepresented in clinical guidelines, which leads to a lack of evidence-based medicine supported results useful for daily practice. In this context, prudent medication selection is a key step in pharmacotherapy selection. There are some tools available that can help in clinical practice, including different medication lists (e.g., Beers criteria, STOPP/START, and guidelines) and pharmacological recommendations.

The participants will learn about general recommendations on medication selection in this population, focusing on general principles on somatic comorbidities treatment, supported by evidence-based data and real clinical pharmacological tools useful for daily practice.

**Disclosure:**

No significant relationships.

